# Association of ocular blood flow and contrast sensitivity in normal tension glaucoma

**DOI:** 10.1007/s00417-021-05235-8

**Published:** 2021-05-21

**Authors:** David Kuerten, Matthias Fuest, Peter Walter, Babac Mazinani, Niklas Plange

**Affiliations:** Department of Ophthalmology, Uniklinik RWTH Aachen, Pauwelsstr. 30, 52057 Aachen, Germany

**Keywords:** Ocular blood flow, Normal tension glaucoma, Contrast sensitivity, Glaucoma

## Abstract

**Purpose:**

To investigate the relationship of ocular blood flow (via arteriovenous passage time, AVP) and contrast sensitivity (CS) in healthy as well as normal tension glaucoma (NTG) subjects.

**Design:**

Mono-center comparative prospective trial

**Methods:**

Twenty-five NTG patients without medication and 25 healthy test participants were recruited. AVP as a measure of retinal blood flow was recorded via fluorescein angiography after CS measurement using digital image analysis. Association of AVP and CS at 4 spatial frequencies (3, 6, 12, and 18 cycles per degree, cpd) was explored with correlation analysis.

**Results:**

Significant differences regarding AVP, visual field defect, intraocular pressure, and CS measurement were recorded in-between the control group and NTG patients. In NTG patients, AVP was significantly correlated to CS at all investigated cpd (3 cpd: r =  − 0.432, *p*< 0.03; 6 cpd: r =  − 0.629, *p*< 0.0005; 12 cpd: r =  − 0.535, *p*< 0.005; and 18 cpd: r =  − 0.58, *p*< 0.001), whereas no significant correlations were found in the control group. Visual acuity was significantly correlated to CS at 6, 12, and 18 cpd in NTG patients (r =  − 0.68, *p*< 0.002; r =  − 0.54, *p*< .02, and r =  − 0.88, *p*< 0.0001 respectively), however not in healthy control patients. Age, visual field defect MD, and PSD were not significantly correlated to CS in in the NTG group. MD and PSD were significantly correlated to CS at 3 cpd in healthy eyes (r = 0.55, *p*< 0.02; r =  − 0.47, *p*< 0.03).

**Conclusion:**

Retinal blood flow alterations show a relationship with contrast sensitivity loss in NTG patients. This might reflect a disease-related link between retinal blood flow and visual function. This association was not recorded in healthy volunteers.

## Introduction

Glaucoma is one of the leading causes of irreversible blindness in the world [1].

Whereas first described in 1857, glaucoma in absence of elevated intraocular pressure, consequently named normal tension glaucoma, remains not sufficiently understood today [2–4]. While often heatedly discussed, ocular hemodynamics are nowadays often accepted as a critical risk factor in glaucoma, particularly in these patients without elevated IOP [5–10]

There are two major issues in the discussion about ocular hemodynamics. On one hand, we are still insufficiently able to measure ocular blood flow directly and rely on different surrogates which are related to ocular blood flow depending on the technique used. Furthermore, we don’t know if recorded ocular blood flow alterations in glaucomatous eyes are primary or secondary in nature [11].

Contrast sensitivity (CS) is important in human vision and substantially affects the level of disability experienced by the patient [12–14].

It was previously reported that retinal ganglion cells significantly contribute to contrast sensitivity and contrast adaptation [15–17]. As glaucoma mainly affects the retinal ganglion cells, significant correlations of decreased GC thickness measured via OCT and CS were reported in glaucoma patients [18].

Furthermore, impairments in CS can be detected particularly early in glaucomatous eyes, even prior to visible retinal nerve fiber layer damage, manifested visual field defects, or a decrease in visual acuity [14, 19, 20]. CS might be a more potent parameter to detect subtle visual disturbances in glaucomatous eyes. A recently published Review highlighted the potential benefits of reliable contrast sensitivity testing in glaucoma for better patient assessment and care [21]. Furthermore, a potential benefit in CS monitoring lies in advanced glaucoma stages, where other structural and functional assessments are not suited to monitor further glaucoma progression [18].

Disturbances in ocular blood flow seem to play a critical role in glaucoma, particularly in normal tension glaucoma (NTG). To date, little is known concerning the association between altered blood flow and visual function in glaucoma. The aim of this study was to investigate if alterations in ocular blood flow are correlated to CS test performance in NTG patients. To verify that this potential relationship (altered blood flow leading to weaker functional performance) only exists in glaucoma patients, a control group of healthy test subjects was recruited.

## Methods

Twenty-five patients suffering from NTG (age 55.23 ± 12.15 years, 5 male and 20 female) and twenty-five healthy control subjects (age 43.59 ± 12.41 years, 12 male and 13 female) were included in this study.

All examinations in this study were performed in accordance with the Declaration of Helsinki for research involving human subjects. Informed consent was acquired from each patient and the study was approved by the local ethics committee (EK 123/03) All participants underwent a detailed ophthalmological examination prior to the inclusion in this study. Patients with best-corrected visual acuity > 0.22 LogMAR (< 0.63 decimal) were excluded from the study. Further exclusion criteria were other significant ocular comorbidities that could either influence ocular blood flow or contrast sensitivity testing (e.g., diabetic retinopathy, macular degeneration, optic nerve atrophy by other causes than glaucoma).

Patients with NTG had glaucomatous excavation of the optic disc (i.e., vertical cup-to-disc ratio > 0.6, or CDR asymmetry > 0.2, or presence of focal thinning or notching in compliance with other studies) and a glaucomatous visual field defect as defined by the European Glaucoma Society. The diagnostic criteria for glaucomatous visual field loss are as follows. Field loss was considered significant when (a) glaucoma hemifield test was abnormal, (b) 3 points are confirmed with *p* < 0.05 probability of being normal (one of which should have *p* < 0.01), not contiguous with the blind spot, or (c) corrected pattern standard deviation (CPSD) was abnormal with *p* < 0.05. All parameters were confirmed on two consecutive visual fields performed with Humphrey Field Analyzer. All patients with glaucomatous visual field loss underwent diurnal curves of IOP measurements (Goldmann applanation tonometry) at 8.00 h, 12.00 h, 16.00 h, 20.00 h, and 24.00 h without any topical or systemic IOP-lowering medication. In patients with NTG, diagnosis was confirmed by readings of IOP never above 21 mmHg. All patients had no other serious eye diseases (e.g., age-related macular degeneration, diabetic retinopathy, and vascular occlusive diseases).

Topical antiglaucomatous eye drops were discontinued and washed out in the NTG group for 3 weeks prior to the inclusion into this study. No topical medication was taken by the healthy control patients.

The CSV-1000 (VectorVision, Greenville, OH) was used for CS measurement. The device measures contrast sensitivity at 3, 6, 12, and 18 cycles/degree frequency. The device projects 4 double rows (rows A, B, C, and D) displaying circles of decreasing contrast sensitivity at 3, 6, 12, and 18 cycles/degree, respectively. Each row consists of 17 circles, with the first circle of each row displaying the highest contrast. The remaining 16 circles are presented in 2 rows consisting of 8 pairs of circles. The patient is instructed to choose the one circle out of each pair showing the grid pattern. The last correct response for each level of contrast is defined as the contrast threshold for that spatial frequency [18].

Systemic and diastolic blood pressure and heart rate were recorded after a resting time of 5 min in the supine position before fluorescein angiography.

Fluorescein angiography with a scanning laser ophthalmoscope (Rodenstock, Ottobrunn, Germany) was performed to evaluate the AVP and for digital image analysis. We previously described the technique in greater detail [22]. A 40-degree observation centered on the optic nerve head was used. At the beginning of the angiography, 10% sodium fluorescein dye (2.5 cc Alcon, Freiburg, Germany) was injected into the antecubital vein. Image acquisition was performed with constant parameters until the maximum intensity level in the retinal veins had passed to avoid artifacts. The dynamic sequences were acquired with 25 images per second. The angiograms were analyzed by digital image analysis (Matrox Inspector, Matrox Inc., Quebec, Canada). The retinal AVP was calculated using dye dilution curve analysis. The AVP represents the shortest passage of the fluorescein dye from the retinal arterioles to the venules representing retinal microcirculation. The intensity level for each image was calculated at a predetermined region of interest (ROI) consisting of the arterioles and venules; the extend corresponded to vessel diameter. All measurements were performed in the temporal superior and inferior arterioles and venules. AVP was calculated by an independent investigator blinded to the study groups, and mean AVP was used for further analysis.

Fisher’s transformation was used to find statistically significant correlations between the individual parameters. Unpaired t-test was used for the analysis of the descriptive parameters in both groups. *p* values were set at 0.05 to be considered statistically significant. Matlab (Version R2018b for Windows) as well as GraphPad Prism (Version 7.0 for Windows) was used for statistical analysis.

## Results

Baseline parameters of the two groups are presented in Table 1.

**Table 1:** Baseline parameters of Healthy Control as well as NTG patients

	NTG	Healthy control	Unpaired T-test
Age	55.2 ± 12.2	43.6 ± 12.4	*p* < 0.0016
IOP	16.0 ± 2.7	14.4 ± 2.7	*p* < 0.03
Visual field mean defect (MD)	− 6.2 ± 5.2	− 0.3 ± 1.1	*p* < 0.001
Visual field PSD	7.2 ± 4.6	1.6 ± 0.4	*p* < 0.0001
Vertical CDR	0.7 ± 0.1	0.4 ± 0.2	*p* < 0.0001
Contrast 3 CPD	1.5 ± 0.3	1.8 ± 0.2	*p *< 0.001
Contrast 6 CPD	1.7 ± 0.3	1.9 ± 0.2	*p* < 0.005
Contrast 12 CPD	1.3 ± 0.3	1.6 ± 0.3	*p* < 0.0001
Contrast 18 CPD	0.9 ± 0.4	1.2 ± 0.2	*p* < 0.0001
AVP	2.3 ± 0.6	1.6 ± 0.5	*p* < 0.0007
Systolic blood pressure	126.3 ± 16.5	130.3 ± 24.2	*p* > 0.49
Diastolic blood pressure	76.4 ± 11.3	81.8 ± 13.9	*p* > 0.14
Heart rate	76.7 ± 11.5	71.7 ± 12.6	*P* > 0.15

NTG = normal tension glaucoma, IOP = intraocular pressure, PSD = pattern standard deviation, CDR = cup-to-disc ratio, CPD = cycles per degree, AVP = arteriovenous passage time

**Fig. 1:**
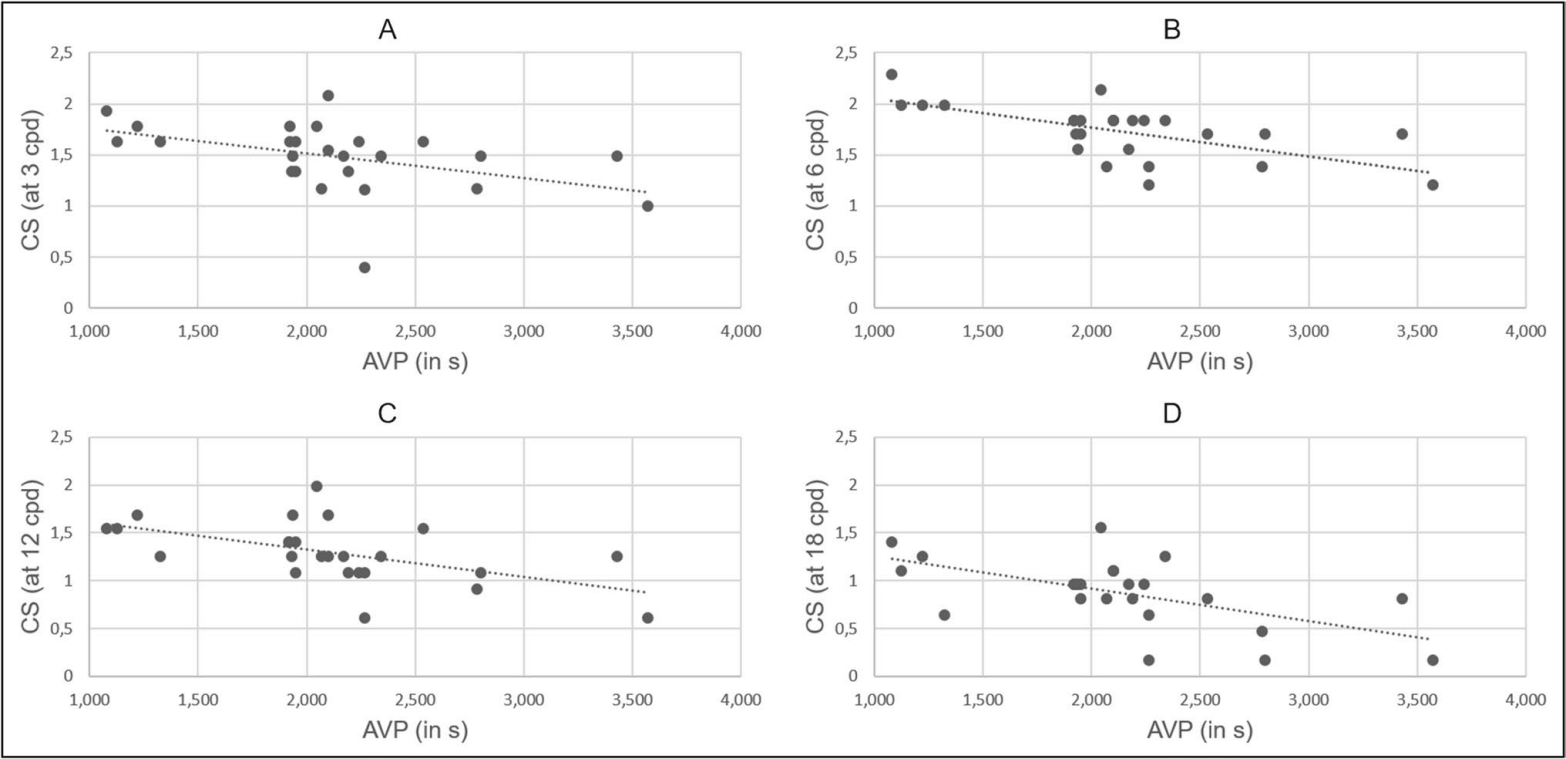
The significant correlations of arteriovenous passage time (AVP) in seconds with contrast sensitivity (CS) testing at 3 cycles per degree (cpd) (**A**), 6 cycles per degree (**B**), 12 cycles per degree (**C**), and 18 cycles per degree (**D**)

For the NTG patients, statistically significant correlations were computed between AVP and contrast sensitivity at 3 cpd (r =  − 0.432, *p* < 0.03), at 6 cpd (r =  − 0.629, *p* < 0.0005), at 12 cpd (r =  − 0.535, *p* < 0.005), and at 18 cpd (r =  − 0.58, *p* < 0.001) using Fisher’s transformation. Please refer to Fig. 1 for visualization.

No significant correlations were computed between AVP and contrast sensitivity in healthy control subjects (r = 0.069, *p* > 0.74; r = 0.36, *p* > 0.07; r = 0.203, *p* > 0.33; r = 0.205, *p* > 0.33 respectively).

In neither NTG patients nor healthy control subjects was age significantly correlated to CPD (*p* > 0.14 in all calculations).

**Fig. 2:**
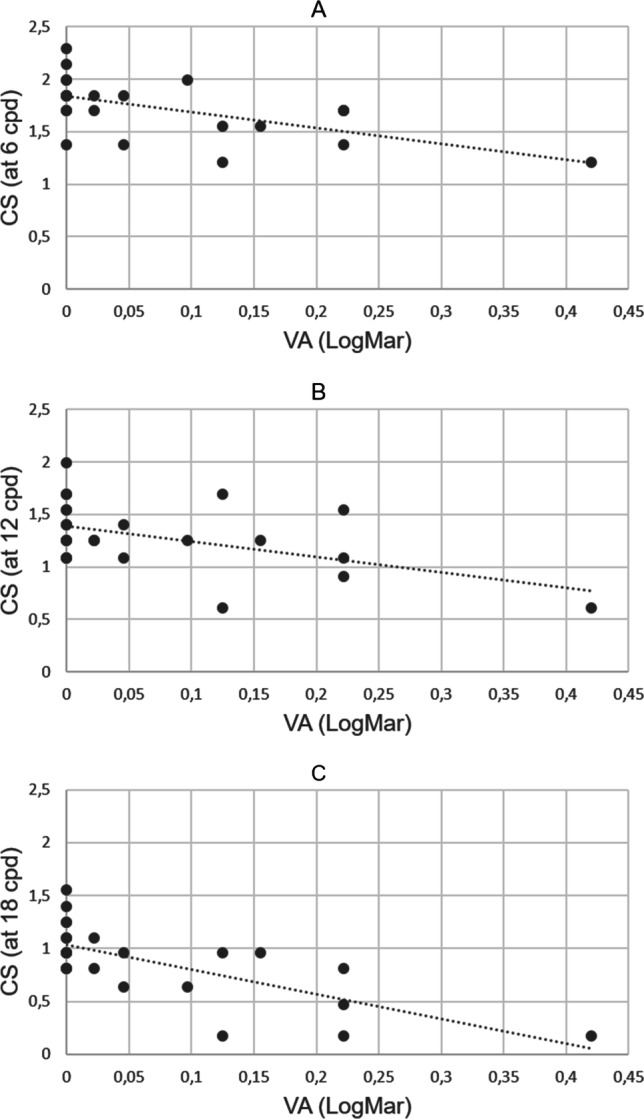
Significant correlations of visual acuity (VA) LogMar and contrast sensitivity (CS) testing at 6 cycles per degree (cpd) (**A**), 12 cycles per degree (**B**), and 18 cycles per degree (**C**)

Visual acuity was significantly correlated to CS at 6, 12, and 18 cpd (r =  − 0.68, *p* < 0.002; r =  − 0.54, *p* < 0.02; and r =  − 0.88, *p* < 0.0001 respectively) (please refer to Fig. 2) as well as AVP (r = 0.69, *p* < 0.002) in NTG patients. Whereas in the healthy control group, visual acuity was only correlated to age (r = 0.56, *p* < 0.009).

MD and PSD were not significantly correlated to CS at any cpd in NTG patients. However, significant correlations were found in healthy patients between MD and PSD and CS at 3 cpd (r = 0.55, *p* < 0.02; r =  − 0.47, *p* < 0.03).

Furthermore, AVP was not significantly correlated to MD, PSD, and age in both groups (*p* > 0.06 always).

## Discussion

The aim of this pilot study was to test our hypothesis that altered ocular blood flow in glaucomatous eyes is correlated to alterations in CS.

Our analysis showed significant correlations between AVP and CS in NTG eyes whereas no such correlations existed in the healthy control subjects. Slower blood flow in glaucomatous eyes (represented in increased AVPs) was significantly correlated to worse performance in CS. No significant correlations were computed for AVP and either MD or PSD in our patients.

Our findings highlight a potential direct link between impaired ocular blood flow and visual function in patients with NTG. Reduced ocular blood flow remains an important issue not only in the pathogenesis of NTG, but also with a direct impact on visual disturbances in these patients.

The findings, significant correlations between blood flow and CS but not VF-performance in glaucomatous eyes, highlight that CS and VF present different functional aspects of the human visual system that are often correlated [19, 23] but different in nature [18]. Interestingly, MD and PSD were correlated to CS at 3 cpd in our healthy control patients, whereas no such correlation was computed in the NTG eyes. Better performance in VF testing (represented in higher MD and lower PSD) was accompanied by better performance in CS testing. As this was only significant at 3 cpd, we are inclined to believe that it might be a result of the small sample size.

The potential importance and benefits of CS testing have recently been highlighted in various studies. Fatehi et al. [18] were able to record significant correlations between central VF summary indices and central macular thickness measurements and CS in glaucomatous eyes. Furthermore, Amanullah et al. [24] were able to record a linear relationship with RNFL thickness. Thakur et al. [25] were able to show that contrast sensitivity scores are associated with the severity of glaucomatous disease. Besides, Lin et al. [26] reported that contrast sensitivity is a vital parameter to predict visual disability in glaucoma.

CS testing provides additional important functional information in glaucomatous eyes that can either be used in the early diagnosis of glaucoma or in advanced stages to monitor further disease progression, whereas the currently available conventional diagnostic tools often fail to provide reliable measurements [21]. In our study, neither MD nor PSD were correlated to CS in our glaucoma patients, supporting the thesis that CS testing might open a new avenue in glaucoma diagnosis and follow-up.

Investigations into the associations of altered blood flow and visual function such as CS parameters are sparse to date.

Harris [27] reported in 1999 that accelerated AVP and improved CS at 3 and 6 cycles per degree were recorded in NTG patients receiving topical dorzolamide. The lack of significant improvement at 12 and 18 cpd was attributed to higher between- and within-subject variability in higher frequencies. We were able to show that baseline alterations in AVP are significantly correlated with all frequencies including the higher ones in NTG patients, strengthening the integral association of ocular blood flow and CS.

Similar findings regarding the effect of dorzolamide were previously reported by our group for brinzolamide, a different antiglaucomatous drug, as well [28]. Thirty healthy test subjects were prospectively randomized to either brinzolamide or placebo during a 2-week double-masked treatment trial. IOP was significantly reduced, CS at 3 cpd significantly increased, and AVP significantly reduced under topical treatment. These results highlight that there is an association between IOP, ocular blood flow, and CS.

In the study, reduced IOP resulted in improved CS; accordingly, Owidza et al. [29] reported that patients with elevated IOP suffering from POAG and OHT show significantly reduced CS. As IOP is integral in ocular perfusion, these findings might be a result of improved or rather reduced blood flow.

The importance of ocular blood flow in glaucomatous eyes has been shown in other studies as well. In previous studies, ocular blood flow disturbances showed a significant correlation to the extent of visual field defects in glaucomatous eyes [30, 31]. Some studies were able to find significant correlations between blood flow disturbances and visual field progression to a certain degree [5, 32]. Furthermore, it was reported that retinal blood flow disturbances are correlated to ocular perfusion pressure in normal tension glaucoma patients [6]. Therefore, disturbances in ocular perfusion pressure due to autoregulatory deficiencies seem to result in retinal perfusion alterations in glaucomatous eyes; these disturbances might be the reason for altered performance in demanding functional tasks such as CS testing.

However, the complexity of the issue is exemplified by the findings of Hosking et al. [33]. The authors showed that mild hypercapnia, which is known to improve ocular blood flow, resulted in a significant fall in contrast sensitivity in previously untreated glaucoma patients and did not alter CS in healthy test subjects. One possible explanation may be that baseline blood flow in glaucoma patients is severely altered, and although ocular perfusion increases due to mild hypercapnia, the increase is insufficient to compensate for increased metabolic stress. On the other hand, disturbed autoregulation in glaucoma eyes might not be able to provide sufficient blood flow to compensate for the increased metabolic stress in the Hosking et al. [33] study.

To further complicate the issue of blood flow and glaucoma, Harris et al. [34] reported that middle cerebral artery (MCA) blood flow was significantly correlated to different functional parameters including CS in glaucomatous patients. Intraocular blood flow alterations were not recorded in this study; therefore, it is not known if MCA blood flow was correlated with intraocular blood flow and subsequently ocular function in these patients.

This and other studies highlight how poorly our understanding of glaucoma is to date.

We and other authors are strong believers that glaucoma is a systemic disease that primarily manifests with ocular damage. We were able to differentiate NTG patients from healthy test subjects just by continuous heart rate and blood pressure measurement with high specificity and sensitivity [35]. These studies support our understanding that blood flow alterations, either systemically and/or locally, play a critical role in glaucoma.

Therefore, in the future, it appears necessary that we ophthalmologists start looking in new directions and even beyond the eye in search of a better understanding of glaucoma.

Some limitations in our work have to be mentioned. The patients in both groups were not matched for systemic disease as well as systemic medication. The exact influences of systemic medication on ocular blood flow have not been described in greater detail today. However, various studies reported partially contradicting results on the influence of topical medication on ocular blood flow [36], and this factor was ruled out in our study, by discontinuing the topical medication in all NTG patients. Furthermore, the groups differed significantly in age as well as gender distribution. While we were unable to compute significant correlations of age with either CS or AVP, the influence on both can’t be ruled out completely and it is known that CS performance is age-dependent and a linear decline in CS was recorded in the age group 50 to 87 years [37]. In addition, systemic diseases such as migraine, arterial hypotension, or Raynaud’s phenomenon might be a part of the concept in NTG-associated ocular blood flow alterations. Not only reduced ocular blood flow but also disturbances in autoregulation or vasospasm might affect glaucomatous optic neuropathy. An association between visual function and blood flow, as shown in our study in NTG but not in healthy subjects, might be a hint to this phenomenon [35].

In summary, this study was able to verify that blood flow alterations in untreated NTG patients are significantly correlated to CS function in glaucomatous eyes, whereas no association exists in healthy test subjects. Further studies are necessary to verify that including CS testing as well as blood flow measurement is beneficial in the assessment and care of glaucoma patients.

## Data Availability

Not applicable.
